# Watching TV news as a memory task -- brain activation and age effects

**DOI:** 10.1186/1471-2202-11-106

**Published:** 2010-08-26

**Authors:** Lars Frings, Irina Mader, Michael Hüll

**Affiliations:** 1 Section of Gerontopsychiatry and Neuropsychology, Medical School, Albert-Ludwigs-University Freiburg, Germany; 2 Center of Geriatrics and Gerontology Freiburg, Albert-Ludwigs-University Freiburg, Germany; 3 Section of Neuroradiology, University Medical Center, Albert-Ludwigs-University Freiburg, Germany; 4 Freiburg Brain Imaging, University Medical Center, Albert-Ludwigs-University Freiburg, Germany

## Abstract

**Background:**

Neuroimaging studies which investigate brain activity underlying declarative memory processes typically use artificial, unimodal laboratory stimuli. In contrast, we developed a paradigm which much more closely approximates real-life situations of information encoding.

**Methods:**

In this study, we tested whether ecologically valid stimuli - clips of a TV news show - are apt to assess memory-related fMRI activation in healthy participants across a wide age range (22-70 years). We contrasted brain responses during natural stimulation (TV news video clips) with a control condition (scrambled versions of the same clips with reversed audio tracks). After scanning, free recall performance was assessed.

**Results:**

The memory task evoked robust activation of a left-lateralized network, including primarily lateral temporal cortex, frontal cortex, as well as the left hippocampus. Further analyses revealed that - when controlling for performance effects - older age was associated with greater activation of left temporal and right frontal cortex.

**Conclusion:**

We demonstrate the feasibility of assessing brain activity underlying declarative memory using a natural stimulation paradigm with high ecological validity. The preliminary result of greater brain activation with increasing age might reflect an attempt to compensate for decreasing episodic memory capacity associated with aging.

## Background

The declarative memory system of the human brain has been a matter of research for a long time. Much knowledge has been acquired by neuropsychological examination of brain-lesioned patients [1,2]. In the last decades, a tremendous input came from neuroimaging studies allowing for the non-invasive investigation of memory [3,4].

The vast majority of experimental studies, however, dealing with the trade-off between well controlled experimental settings and real-life stimuli favored laboratory stimuli whose reduced complexity is typically dissimilar from what the human brain encounters in everyday life.

Only recently, a few brain mapping studies have successfully used movies as 'natural' stimuli to assess brain responses reflecting processing of stimulus properties [5-7]. We argue for a principle advantage of natural, ecologically valid stimuli over typical, rather artificial laboratory stimuli: Examining cognitive function with rather natural stimuli creates a more comfortable setting for participants, which might decrease anxiety effects on the test performance and lead to greater tolerability - a crucial point when it comes to assessment of patients with severe cognitive deficits like in dementia. Furthermore, while functional segregation of the cerebral cortex is best assessed using stimuli with simplified, abstracted features, many brain areas deal with multimodal integration of complex stimulus arrays, processing complex situations simultaneously and interactively. Higher-level, multimodal integration areas might be best tackled by complex, natural stimulation.

Solid grounds have been prepared by basic memory research in the last decades, which now offers the opportunity to target how information is acquired during more complex, real-life stimulation. In contrast to a recent study investigating memory processes with fMRI during presentation of a TV sitcom [7], the current study aimed at testing brain activation during a prototypical example of everyday life information acquisition: watching TV news. This has, to the authors' knowledge, not been the subject of brain imaging research before.

A secondary aim of the current study was to assess the effects of age on brain activation during information acquisition. Research of brain/behavior relationships in healthy aging has recently gained much attention. While cognitive performance differences between younger and aged adults are well established in some domains, e.g., episodic memory, cognition in other domains does not seem to be affected by aging, e.g., semantic memory [8]. Despite obvious morphological changes and concomitant decrease of brain capacity in later life [9], the healthy aging brain performs remarkably well. It has been hypothesized that compensatory mechanisms sustain performance despite erosion of resources [10].

In this study, we aimed at testing whether (1), an fMRI paradigm using natural, real-life stimuli would elicit activation of the declarative memory system, and (2), whether using this paradigm allows for the detection of age-related differences in brain activity related to the task.

## Methods

### Participants

Seventeen neurologically healthy subjects (9 female, 8 male) participated in this study. All except for one female participant were right-handed as assessed with the Edinburgh Handedness Questionnaire. We included this left-handed participant in the current analyses after confirming that her brain activation pattern during the language-dependent task described below was as left-lateralized as those of the remaining participants. This was regarded as evidence for left-hemispheric language-dominance in all participants. Age ranged from 22 years to 70 years (Mean (SD) = 41.5 (18.0) years). Splitting the age range at the group mean, three females and four males were older, six females and four males were younger. Distribution across the age range was not statistically different between the sexes (Chi-Square-Test). Formal education was 11 years or above in 16 participants, it was not assessed in one participant. Standardized cognitive test data were acquired from participants above 55 years of age with the Mini-Mental State Examination (MMSE [11]), in order to exclude undiagnosed demented subjects; MMSE scores were 27 or higher. Written informed consent was obtained from each participant. The study was performed according to the Declaration of Helsinki of 1964 and approved by the Ethics Committee of the University of Freiburg.

### Memory Task

As stimuli presented during MR scanning we used clips of the most famous German daily TV news show 'Tagesschau' from the ARD channel, which were taken from video podcasts publically available on the internet (http://www.tagesschau.de). Six clips were chosen, each comprising a complete news story in 20-30 seconds that was read by a visible female or male speaker with a picture in the background presenting a related figure or photograph together with a related headline. News regarded as especially arousing were excluded. At the time of scanning, news clips were more than 1 year old. None of the participants later reported remembering the original TV broadcasting of the video clips.

As a control condition we used rearranged versions of the same news clips with the audio track reversed: We used Matlab7 (http://www.mathworks.com) to divide each image of the video clip into 80 × 80 equal rectangles, each of 4 pixels width and 3 pixels height. Rectangles were randomly rearranged, such that pictures, speakers, or faces were not recognizable. Thus, the physical properties of the pixels as well as the sound track were widely retained from the original, while lacking meaningfulness.

Additionally, a fixation cross baseline condition was randomly interspersed. Duration of blocks was identical between conditions. Block presentation was randomized before the first subject was scanned. Presentation order was held constant across participants.

Prior to the experiment participants were instructed to listen and watch carefully, and to keep in mind as much of the presented news clips as possible, as it would be asked for detailed contents afterwards. During a test trial in the scanner prior to the experiment, each participant confirmed good hearing and sight of the audio and video stimuli. Audio was presented via an MR-compatible headphones system (MR-Confon GmbH, Magdeburg, Germany), which in addition to scanner noise insulation allowed for individual volume adjustment. No participant requested volume elevation of > 2 dB from default volume. After scanning, i.e., after a delay of about 10 minutes due to structural image acquisition and leaving the scanner room, the participant was asked to recall the six news clips in as much detail as possible. We took the percentage of correctly recalled content words as a measure of free recall performance. As such, the task and its scoring resemble the logical memory subtest from the revised Wechsler Memory Scale.

### MR Imaging

During the memory paradigm (<8 minutes), 208 BOLD-sensitive echo planar images of the entire cerebrum were acquired using a 3 Tesla Siemens TIM-Trio (Siemens, Erlangen, Germany). We used a BOLD-sensitive EPI-sequence with TR/TE = 2190/30 ms (36 contiguous axial slices of 3 mm thickness covering the entire cerebrum, in-plane resolution 3 × 3 mm; flip angle 75°) and automated online motion and distortion correction with an in-house software [12]. Afterwards, a T1-weighted MPRAGE (TR/TE = 2200/2.15 ms, 12° flip angle, 1 mm isotropic voxel size) was acquired which was used as a structural reference for processing of the functional images.

Preprocessing and analyses of the imaging data were performed with SPM5 (Wellcome Trust Centre for Neuroimaging, Institute of Neurology, UCL, London, UK -- http://www.fil.ion.ucl.ac.uk/spm). The MPRAGE image of each participant was segmented into GM, WM, and CSF, using the unified segmentation procedure of SPM5. Resulting normalization parameters were used for warping structural and functional images into MNI-space, functional images were resampled to 3 mm isotropic voxel size and smoothed with an 8 mm isotropic Gaussian kernel. Single-subject statistics involved modeling of each condition plus 6 realignment parameters and contrasting of BOLD signal during clip vs. rearranged clip presentation. The contrast image representing the contrast 'clip > rearranged clip' was taken to 2^nd ^level, random effects group statistics. We performed a One-sample T-test in order to determine a group activation map reflecting visual and auditory higher-order perception (in contrast to meaningless auditory and visual stimulation in the control condition) and declarative memory processes. Results were thresholded at the voxel-level at p < 0.001, false discovery rate (FDR)-corrected for multiple comparisons. Only clusters larger than seven voxels were reported, corresponding to p < 0.05, uncorrected at the cluster-level. Additionally, in order to investigate the relation between regional activation and performance, individual activation strength at peak voxels of the group activation map, together with individual free recall performance, were subjected to a Pearson correlation analysis.

Further, a multiple regression was performed in order to seek for brain responses in relation to age. Regressors were included coding for sex, age, and performance. As the effect of primary interest was age, performance and sex effects were controlled for. The results of this analysis were thresholded at a less conservative threshold (p < 0.001, uncorrected) in order not to overlook the effects of age, which we assumed to be more subtle than the main effect of the task. Only clusters larger than seven contiguous voxels were reported.

## Results

### Behavioral Data

As expected, all participants were well able to perform the task. All denied excess exhaustion, which we attribute to the very short duration of the scanning protocol (8 min task plus 7 min structural scanning). Mean memory performance over participants was 22% (SD = 7%) correctly recalled items. Memory performance exhibited a highly significant, negative correlation with age (Pearson r = -.7; p < 0.01). Older participants showed rather constant recall across the six news clips: the standard deviation of % recalled items over clips showed a tendency towards a significant, negative correlation with age (r = -.45; p < 0.075).

### FMRI Task Effect

**Figure 1:**
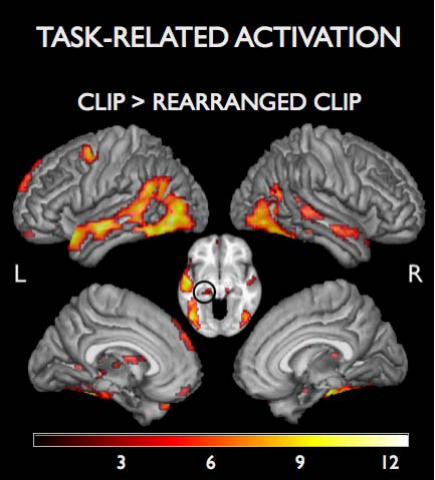
**Task-related group activation (p < 0.001, FDR-corrected), overlaid on a group average T1-weighted image in MNI space. **Circle on the axial slice highlights hippocampal activation. Color scale indicates T-values.

The one-sample T-test at the group-level revealed a left-lateralized activation pattern which involved mainly a large cluster in the left temporal lobe (p < 0.001, false discovery rate (FDR)-corrected for multiple comparisons). Its peaks were in the temporal pole (TP), middle temporal gyrus (MTG), inferior occipital gyrus (IOG), and in the left hippocampus. In the right hemisphere, activated regions included portions of the temporal lobe (primarily fusiform gyrus, MTG, medial TP) and inferior parietal cortex. For further activated regions, see Table 1 and Figure 1.

**Table 1:** Clusters of voxels significantly activated by the task.

Hemisphere	Anatomical Region	Cluster Size (Voxel)	Peak MNI Coordinates	Peak T-Score
L	temporal pole; MTG; IOG; hippocampus	1263	-54 12 -21	12.16
R	fusiform gyrus; IOG; MOG; MTG	344	42 -45 -21	12.55
R	MTG; STG	83	54 -36 0	7.23
R	MTG; medial temporal pole	68	57 3 -18	8.24
L	precentral gyrus	52	-39 -3 51	8.52
R	Inferior parietal cortex	29	36 -63 18	7.11
	cerebellar vermis	25	3 -66 -27	6.67
L	SFG	23	-9 51 30	6.03
L	rectal gyrus	13	-3 51 -15	6.07
L	thalamus	9	-15 -6 12	6.07

One-sample T-test of the contrast 'clip > rearranged clip', thresholded at p < 0.001 (FDR-corrected).

In order to test whether greater activation was related to better memory performance, additional correlation analyses were performed. Individual activation strengths (in terms of the T-values from the single subject analyses) were extracted from those voxels which exhibited a highly significant main effect of task (Table 1) plus two further local maxima within the largest cluster: left MTG [-54 -18 -9] and left hippocampus [-18 -27 -9]. A significant relation between better performance and greater activation was observed in the right fusiform gyrus ([42 -45 -21], Pearson correlation coefficient r = .67; p < 0.01), left precentral gyrus ([-39 -3 51], r = .55; p < 0.05). A tendency towards a significant relationship was revealed in the left MTG ([-54 -18 -9], r = .41; p = 0.055, one-tailed).

### FMRI Age Effect

**Figure 2:**
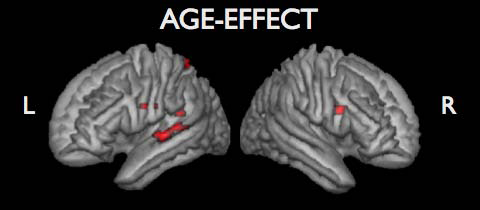
**Pattern of voxels showing an association between greater task-related activation and older age (p < 0.001, uncorrected)**.

The multiple regression analysis showed a significant relation between older age and greater activation - controlling for sex and performance effects - in left lateral temporal cortex and the left superior parietal lobule. In the right hemisphere, a cluster in the frontal lobe - corresponding to BA 44 according to the probabilistic atlas of Eickhoff et al. [13] - showed an association with age (Table 2; Figure 2). A significant association between older age and *decreased *activation was not observed.

**Table 2:** Clusters of voxels which exhibited a significant relation between greater activation and older age (multiple regression, p < 0.001, uncorrected).

Hemisphere	Anatomical Region	Cluster Size (Voxel)	Peak MNI Coordinates	Peak T-Score
L	STG; STS	28	-63 -15 0	5.55
L	superior parietal lobule	16	-30 -60 60	5.65
R	BA 44	11	42 6 21	4.82

## Discussion

By using clips of a TV news show as stimuli in an fMRI experiment, we were able to demonstrate brain activation in healthy participants across a wide age-range that involved the medial and large portions of the lateral temporal lobe - brain regions which have previously been ascribed a crucial role in episodic memory and semantic processing, respectively.

Further, greater activation in left temporal and right frontal cortex was related to older age. Thus, the paradigm applied in this study was not only apt to elicit activation within the declarative memory system, but also revealed a more subtle effect of age-related changes in brain activation during this natural stimulation task. This suggests that research of healthy aging and its alteration in degenerative diseases like dementia of Alzheimer's type might benefit from natural, real-life experimental paradigms.

Natural stimulation paradigms, especially when assessing healthy elderly and demented patients, might be better tolerated by participants as compared to more artificial task settings with rather complex task instructions, and hence, in principle, increase validity of results.

We regard our activation condition as a prototypical example of 'natural stimulation'. This does not refer to similarities between watching TV and real-life interaction with another person. Rather, it refers to watching TV in our culture being one of the core situations designated for information acquisition. As such, TV watching itself has become 'natural' stimulation.

The typical drawback of experiments using natural stimulation as compared to artificial laboratory stimuli lies in the less controlled nature of the cognitive processes evoked. This is especially true for complex, multimodal stimulation. We tried to rule out confounds by contrasting with a control condition which very closely matched to the experimental condition while removing meaningfulness. In fact, control condition stimuli were derived from the exact counterparts of the experimental condition, with the visual presentation rearranged and audio reversed. We thus contrasted brain activity (regional BOLD response) during an episodic memory task, which required semantic processing, to a control condition without semantic or episodic memory processing. This led to almost identical activation patterns when contrasting each of the two with the fixation baseline -- predominant activation of primary visual and auditory cortex. Intensity of visual features was, however, not absolutely matched in the control condition. Coherent motion, e.g., was rather attenuated in the control condition, faces were not recognizable, and more sharp edges were introduced due to frame scrambling. Therefore, differences between experimental and control conditions in early visual areas might be enhanced and not explained by declarative memory. Similarly, while reversing speech (rendering it incomprehensible) reduces semantic processing, at the same time phonological and syntax processing might as well be reduced.

### Main Task Effect

The main effect of the memory task (clip > rearranged clip) presumably reflects language processing in lateral temporal cortex, memory encoding in the hippocampus, processing of visual features occipito-temporal cortex, and multimodal integration in the STS.

We assume the observed left-lateralized temporo-lateral activation to correspond largely to the ventral stream brain regions which have been demonstrated to be crucially involved in language processing [14,15]. Dorsal stream regions seem to be less engaged in the task, presumably due to high demands on input (comprehension) in contrast to output processing (articulation). The areas involved include the left temporal pole, which has been termed the 'semantic hub' [16] as it is implicated in semantic processing [17,18]. The inferior, posterior temporal activated region presumably corresponds to a sound-meaning interface, a function which has been ascribed to this region [14] that has also been termed the 'basal temporal language area' [19]. Further activated spots along the long axis of the MTG map nicely onto what has recently been termed the large-scale semantic network of the human brain [20]. We thus assume that much of the observed left lateral temporal cortical activation pertains to semantic processing of incoming information, which is a prerequisite for episodic memory formation provided by the hippocampus [21]. The crucial role of the hippocampus for episodic memory formation and, in part, retrieval is well characterized in the literature [2]. This suggests that the observed hippocampal activation reflects episodic memory-related processing.

The link between memory task-related activation patterns and declarative memory processing is corroborated by the finding that stronger task-related activation was related to better performance in temporal and frontal cortex (see Results).

It has previously been demonstrated that complex, multimodal stimuli, like watching a sequence of a movie, are apt to detect activity of several cortical regions which are specialized for unimodal features like motion, color, or faces [5]. The task-related activation pattern in the current study as well includes cortical regions which are specialized for processing unimodal features like motion (in the occipito-temporal junction, presumably V5), or faces (in the posterior fusiform gyrus; c.f. Figure 1). Beyond signal responses to unimodal stimulus features, the main task effect in the current study as well evoked activation of multimodal integration cortex, the STS [22].

### Age effects

Brain regions which exhibited an increased task-related activity with older age were observed in left temporal, frontal, and posterior parietal cortex, as well as in a right frontal region, corresponding to BA44. This increased BOLD response in the latter region might reflect a compensatory mechanism in terms of recruitment of an additional contralateral homologue of the left hemisphere Broca area for language comprehension, as has been observed in left hemisphere stroke patients [23]. Likewise, increased frontal activity with age has recently been described [24], though coupled to a decrease in occipital cortex, which we did not see in our data. It might as well point to a reduced hemispheric asymmetry in older age, which has previously been hypothesized [25]. Alternatively, frontal cortex involvement in elderly participants might as well reflect a reduced inhibition of an articulatory network [26], which, although tapped by speech input, is rather irrelevant for task completion.

The observed greater activity concomitant with older age in left superior temporal regions -- neighboring those areas recruited by the task -- might be driven by increased effort, which elderly participants needed to perform as good as younger participants (the regression analysis controlled for performance effects). Left mid--part superior temporal cortex has recently been associated with semantic processing [27].

Furthermore, a left posterior parietal region -- close to the intraparietal sulcus -- displayed greater activity in the elderly. This might reflect increased involvement of the dorsal attention network [28] or, more specifically, attention to memory [29,30], which was not required for successful task completion in younger participants.

A theoretically possible cause of the observed age-effect on activation might be altered hemodynamics in elderly participants, unrelated to cognition. In healthy elderly, a decline of cerebral blood flow has been shown recently [31], as well as reduced task-related fMRI BOLD signal [32]. In contrast, in our study an increased BOLD response with increasing age was observed, which suggests a neural rather than vascular cause of the observed effect.

It was attempted to rule out contribution of impaired hearing or vision in elderly participants to age-related activation effects. Each participant confirmed good hearing and vision of stimuli before the experiment started. However, as this was not formally tested, it should be considered a possible confound in this analysis.

Activation *decrease *associated with increasing local gray matter atrophy, has been reported previously [33]. The fact that this was not observed in our study suggests that healthy elderly participants did not have sufficient atrophy to reduce the BOLD signal in any region.

In contrast to the robust main task effect, the observed age-effect is considered a secondary, preliminary result of the current study that requires replication by studies assessing larger numbers of participants.

## Conclusion

With the current study we present a natural stimulation fMRI paradigm which robustly activated semantic and episodic memory processing brain regions. As it was apt to detect altered processing in the healthy aging brain in our study, it might prove valuable in the further characterization of pathological aging, e.g., degenerative dementia.

## Authors' contributions

LF designed the study, acquired and analyzed data, and drafted the manuscript. IM contributed to MR data acquisition as well as discussion and revision of the manuscript. MH participated in the design and coordination of the study, and contributed to discussion and revision of the manuscript. All authors read and approved the final manuscript.
